# Automated Detection of Motion Artefacts in MR Imaging Using Decision Forests

**DOI:** 10.1155/2017/4501647

**Published:** 2017-06-11

**Authors:** Benedikt Lorch, Ghislain Vaillant, Christian Baumgartner, Wenjia Bai, Daniel Rueckert, Andreas Maier

**Affiliations:** ^1^Pattern Recognition Lab, Friedrich-Alexander University Erlangen-Nürnberg, 91058 Erlangen, Germany; ^2^Biomedical Image Analysis Group, Imperial College London, London SW7 2AZ, UK

## Abstract

The acquisition of a Magnetic Resonance (MR) scan usually takes longer than subjects can remain still. Movement of the subject such as bulk patient motion or respiratory motion degrades the image quality and its diagnostic value by producing image artefacts like ghosting, blurring, and smearing. This work focuses on the effect of motion on the reconstructed slices and the detection of motion artefacts in the reconstruction by using a supervised learning approach based on random decision forests. Both the effects of bulk patient motion occurring at various time points in the acquisition on head scans and the effects of respiratory motion on cardiac scans are studied. Evaluation is performed on synthetic images where motion artefacts have been introduced by altering the *k*-space data according to a motion trajectory, using the three common *k*-space sampling patterns: Cartesian, radial, and spiral. The results suggest that a machine learning approach is well capable of learning the characteristics of motion artefacts and subsequently detecting motion artefacts with a confidence that depends on the sampling pattern.

## 1. Introduction

Despite producing excellent soft tissue contrast images, the acquisition of an MR scan usually takes longer than subjects, and in particular patients, can remain still. In fact, the scanning time is far longer than most types of physiological motion such as involuntary bulk movements and cardiac and respiratory motion [1–3], as well as the flow of blood [4].

Motion artefacts occur as effects of motion during the acquisition and appear as ghosting, blurring, and smearing. With living subjects it is inevitable to have artefacts to some degree in the resulting images [5]. Image artefacts degrade the image quality and can challenge the diagnostic value of an image, sometimes requiring repeating the scan.

The ability to detect motion artefacts in MR scans could be employed for a multitude of applications. In a clinical context, motion artefact detection would allow a clinician to have real-time feedback on whether a scan should be repeated while the patient is still in the scanner, reducing the necessity to reinvite the patient if the image quality is found to be insufficient due to motion artefacts. To go one step further, artefact detection could be integrated into the machine to automatically decide whether it is necessary to repeat the scan without human interaction and without requiring a specialist to check the quality of the scan. Furthermore, in research, where advanced reconstruction methods in development can take hours or days of time and resources, a method to detect motion artefacts could be employed to make sure at an early stage of the reconstruction process that the effort of an expensive reconstruction is worth doing on the acquired data [6].

The goal of this work is to explore the applicability of a machine learning approach to detect motion artefacts from MR images.

The contributions of our work are as follows:

- We propose a strategy to introduce synthetic motion artefacts in *k*-space.
- We show how a machine learning based approach is capable of detecting motion artefacts and explain its limitations on the given data.

While many studies which use images with synthetic motion artefacts such as [7] focus on the Cartesian acquisition, we also compare synthetic motion artefacts simulated with radial and spiral sampling geometries. As this work is supposed to be a proof of concept, we favored simulating motion artefacts in *k*-space rather than simulating a motion-corrupted raw MRI signal using tools such as the Physics-Oriented Simulated Scanner for Understanding MRI (POSSUM) [8].

Most of the patient movement-related artefacts propagate over the image and into the background. Hence, previously proposed methods for automatic quality assessment in 3D structural MRI such as [9] build on the analysis of the background intensity distribution. After segmenting artefactual voxels in the background region, the authors calculate quality indices in the segmented areas. As a consequence, the method is only applicable to scans with large regions of no intensities, whereas this work's approach is not limited to specific body regions and does not make any assumptions on the intensity distribution in the reconstructed image.

After outlining the MR acquisition and reconstruction, we present our strategy to introduce synthetic motion artefacts in Section 2. In Section 3, we show examples of the effects of motion on the reconstructed slices and discuss the results of automatic motion artefact detection.  Section 4 concludes the paper with a summary and outlook.

## 2. Materials and Methods

To simulate motion artefacts on reconstructed MR scans, we inverted the reconstruction by going back into *k*-space, altering the *k*-space samples in a way that simulates subject movement, and reconstructing the altered *k*-space to create an artefact-corrupted MR scan. All experiments were conducted in 2D. As we will show in the following, this is sufficient to get realistic motion artefacts in the images.

### 2.1. MRI Acquisition

During the acquisition, the object to be imaged is sampled in frequency space, and the recorded signals are stored in *k*-space. *k*-space samples close to the center of *k*-space contribute to low-frequency content and make up the smooth parts of the reconstructed image, whereas data samples further away from the center represent high frequencies and contribute to the edges of the image.

Three commonly used sampling patterns to fill *k*-space are Cartesian sampling, radial sampling, and spiral sampling. With Cartesian sampling, *k*-space is filled line by line. Motion artefacts typically form in the phase-encoding direction, as the time span between two phase-encoding steps is significantly longer than the time span for frequency encoding [10]. As most of the energy is concentrated in the low frequencies that are located close to the center of *k*-space, the Cartesian sampling pattern is particularly sensitive to subject movement during the acquisition of the central lines of *k*-space. Other sampling geometries such as radial sampling or spiral sampling overcome this sensitivity by recording both low-frequency content and high-frequency content in each phase-encoding step such that inconsistencies would be averaged over the number of steps. Such nonequispaced sampling geometries can achieve shorter acquisition times and a better signal-to-noise ratio at the cost of a more difficult reconstruction.

### 2.2. Bulk Head Motion

One of the most frequent sources of artefacts in MRI head imaging is bulk patient motion [3, 11]. Bulk patient motion occurs when a patient moves suddenly. We simulated bulk head motion on a set of T2-weighted MR scans taken from the IXI dataset consisting of 578 volumes from normal, healthy subjects [12]. Each volume contains 130 axial slices of a brain surrounded by background of almost no intensities.

Bulk movement was simulated as rigid motion with six degrees of freedom, comprising three rotation angles and a 3D translation vector.

**Figure 1:**
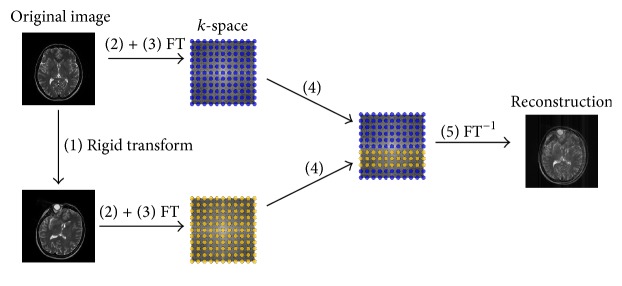
Strategy to introduce bulk motion artefacts with a Cartesian trajectory. The circle-shaped object in the result of the rigid transform is no artefact but one of the eye balls which is acquired in the central slice after the 3D rotation.

The method to introduce motion artefacts from bulk head motion is shown in Figure 1:

1. Transform the original volume rigidly.
2. Extract the central slice of both the original volume and the rigidly transformed volume. The subsequent steps are all working on 2D images.
3. Transform both original and rigidly transformed images into *k*-space. With a Cartesian trajectory, this transform is the fast Fourier transform (FFT). With a radial or spiral trajectory, this transform is implemented in the nonequispaced fast Fourier transform's (NFFT) forward operation.
4. Merge the *k*-space of the original volume and the *k*-space of the rigidly transformed volume by exchanging specific data samples from the original *k*-space with the corresponding data samples of the rigidly transformed *k*-space, producing the “joint” *k*-space.
5. Reconstruct the final image from the joint *k*-space. Due to the inconsistencies that were created in *k*-space, the resulting image is expected to show image artefacts. In case of a Cartesian trajectory, this step is implemented by the inverse fast Fourier transform; for nonequidistant sampling patterns, the NFFT's adjoint operation can be used.

Note that, in case of a radial or spiral sampling pattern, a certain number of radial spokes or spirals, respectively, instead of lines are taken from the *k*-space of the rigidly transformed image. The range for the 3D translation and the three rotation angles were determined heuristically by looking at a surface plot of the head volumes and determining a rotation that seemed natural. The effective values were then chosen randomly within that range with a uniform distribution. The origin of the object was translated up to a maximum of 3.64 mm.

### 2.3. Respiratory Motion on Cardiac Scans

Particularly medical screenings of cardiac or abdominal regions have to deal with periodic motion arising from cardiac activity and respiration [13]. With cardiovascular diseases being a leading cause of death [14], many studies focus on cardiac imaging. In particular 2D imaging sequences can be subject to interslice shift caused by different breath-hold positions from one slice to the next [15].

We synthetically generated artefacts from respiration based on 145 3D scans that were acquired at Hammersmith Hospital as part of the UK Digital Heart project. Since 3D imaging only requires a single breath-hold, the chance that these images contain motion artefacts from respiration is low. Since all data was acquired with the same system, it is safe to assume that the classifier will not learn the characteristics of the system.

McLeish et al. investigated the effects of respiratory motion on the heart by quantifying the motion at a number of points at the right coronary artery, the right atrium, and the left ventricle at different positions in the breathing cycle [16]. According to their studies, rigid-body motion of the heart takes place primarily in the craniocaudal direction with smaller displacements in the right-left and anterior-posterior directions. Typical deformations ranged from 3 to 7 mm. Based on this study, respiration was simulated by translating the heart in the Hammersmith dataset towards the superior direction by up to 7 mm. Segmentations of the ventricular cavities and the myocardium were available from [17].

To simulate various numbers of breathing cycles, we chose a sinusoidal model for respiration. To imitate a subject that completed, for example, four breathing cycles within one acquisition with 256 phase-encoding steps, we sampled a sinusoidal curve with four cycles at 256 time points. A sampled point corresponds to one position in the breathing cycle; that is, a point at the bottom of the sinusoidal curve corresponds to the subject at full inhale, whereas a point at the top of the sinusoidal curve corresponds to the position in the breathing cycle at full exhale. The strategy to generate motion artefacts synthetically closely follows the one described for bulk motion. For periodic motion, however, the subsequent lines, spokes, or spirals, respectively, in *k*-space came from different images depicting the subject at subsequent positions in the breathing cycle according to the sinusoidal model.

### 2.4. Feature Extraction

In total, four types of intensity-based features were computed on each image as input to the classification. The region of interest in which features were extracted from the cardiac data was confined to a rectangular region around the heart. To make sure that this region had the same size for all subjects, the maximum width and height required to fit all subjects were determined and enlarged by 10% in horizontal and vertical direction in total.

The types of features included the following.

*Box Features*. Mean intensity and variance were calculated inside a patch-shaped region with random edge length and position.

*Line Features*. After choosing a line with random starting point and length within a certain radius, the standard deviation and the difference between maximum intensity and minimum intensity along the line profile were taken as two features.

*Histogram Features*. The idea to use histogram-based features was based on the* Autofocus* algorithm for automatic correction of motion artefacts in MR imaging [18]. This motion correction algorithm minimizes the entropy that can be calculated from a histogram. For blurring and smearing into regions of almost no intensities, motion artefacts are expected to decrease contrast and increase entropy.

Histogram features were computed with a random number of bins, some on the whole region of interest and some in patch-shaped regions similar to the box features. From the histogram, six scalar values were calculated: mean intensity, variance, skewness, kurtosis, entropy, and energy. Note that entropy-based features are invariant to rotation when taking the whole region of interest into account. This property is ruled out when only considering patch-shaped regions because of their location.

*Texture Features*. Haralick features were employed to describe the texture characteristics of an image. Adjacency can be defined in four different ways in 2D (horizontal, vertical, left, and right diagonals); thus these texture features differ depending on which of the four directions is considered [19]. The first 13 features proposed in [20] were computed for each of the four directions and the resulting features were averaged over the four dimensions to achieve rotation invariance.

### 2.5. Machine Learning and Evaluation

We used nested cross validation to train a decision forest on the two classes of motion-corrupted and artefact-free images. It was ensured that no subject was included more than once to prevent the classifier from learning characteristics of a particular subject's anatomy. For each subject, either the artefact-free or the artefact-degraded image was picked randomly. Hence, in total, the two classes were represented in equal proportion. The classification accuracy was determined by comparing the predicted labels to the actual labels.

## 3. Results and Discussion

### 3.1. Examples of Bulk Motion Artefacts

Bulk patient motion was simulated as described in Section 2.2 on head scans with different “levels” of artefacts. These levels vary in the time point in the acquisition when the subject moved and the time span in which the subject remained in the other position. The changes in *k*-space can be described by a combination of the fraction of *k*-space exchanged and its offset from the beginning of the acquisition. In a real-world scenario, a smaller fraction corresponds to faster movement of the subject.

**Figure 2:**
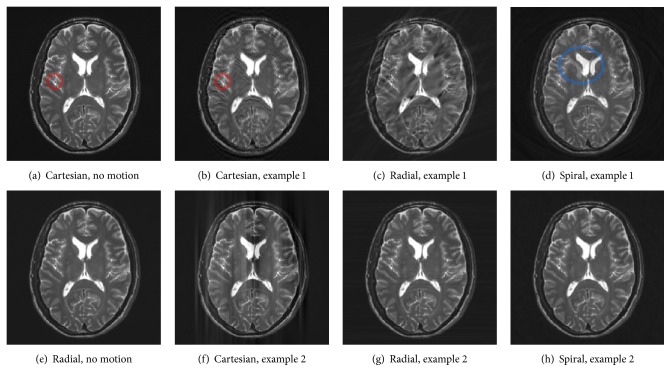
Comparison of bulk motion artefacts acquired with different sampling patterns.

Figure 2 shows examples of how bulk motion artefacts are expressed with different sampling geometries. The scans in Figures 2(a) and 2(e) were simulated with Cartesian and radial sampling patterns, respectively, and without any motion. There are no obvious differences. The same holds for a spiral acquisition without any motion. This was expected and demonstrates that changing the acquisition strategy does not introduce artefacts.

The other images in each row were all acquired with the same amount of motion. The remaining images in Figures 2(b), 2(c), and 2(d) simulated a subject that was moving his head to another position after 20% of *k*-space had already been acquired and moved back to the original state after filling another 20% of *k*-space. With a Cartesian sampling pattern as seen in Figure 2(b), artefacts formed in the phase-encoding direction from top to bottom. Small detail structures like the small dark blob in Figure 2(a) (red ellipse) were blurred and particularly edges of high contrast were replicated. In contrast, the scan acquired with a radial sampling pattern in Figure 2(c) exposes some blurred streaks in diagonal direction. Note that this artefact is very similar to CT imaging [21]. The direction of these streaks depends on which radial spokes in *k*-space are affected by the patient movement and thus the time point in the acquisition. However, small detail structures remained sharp, though some became overspread by streaking. The spiral scan in Figure 2(d) also preserved small detail structures. Nevertheless, a duplicate of the lateral ventricle can be found as clear evidence of ghosting artefacts (blue ellipse).

The examples in Figures 2(f), 2(g), and 2(h) simulated a very sudden movement that lasted 2/256 of the total acquisition time and occurred after half of the scan had completed. In case of Cartesian sampling as seen in Figure 2(f), this movement affects two central lines in *k*-space, leading to low-frequent smearing in the phase-encoding direction. The scan acquired with a radial trajectory in Figure 2(g) again shows some streaking in the horizontal direction which hardly becomes apparent due to the robustness of the sampling pattern to little movement. The streaking would become more apparent if more radial spokes in *k*-space were affected. In Figure 2(h), no artefacts can be seen from visual inspection, demonstrating the forgivingness of the spiral sampling pattern to few inconsistencies in *k*-space.

In contrast to the Cartesian acquisition, it becomes obvious that with radial or spiral sampling patterns the strength of motion artefacts does not or hardly depends on the time point in the acquisition at which the subject movement occurs. This was expected, since both nonequispaced sampling geometries acquire the center of *k*-space with each spoke or spiral, respectively. As an effect, inconsistencies in the low-frequency content are averaged and both sampling patterns become more robust to subject motion.

### 3.2. Classification Results of Bulk Motion Artefacts

Depending on the portion of *k*-space to be replaced, the classification accuracy ranged from 75.4% to 100% of correctly classified images. Only box features were used for training and testing.

**Figure 3:**
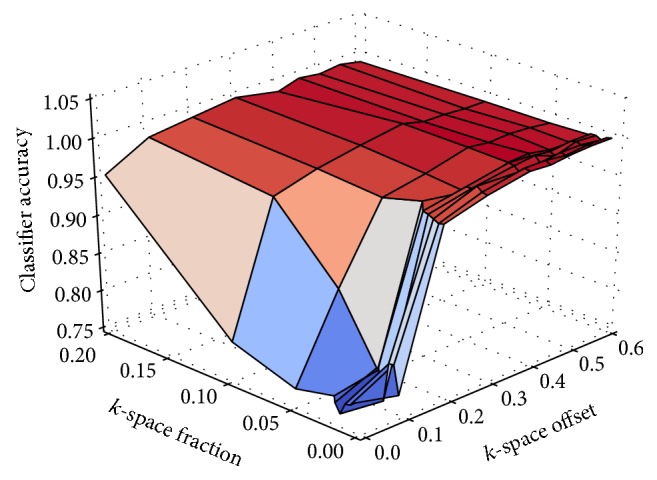
Classification accuracy compared to the size and the position of the fraction that was exchanged in *k*-space with a Cartesian sampling geometry.

Figure 3 compares the classification accuracy on images from a Cartesian acquisition with their level of artefacts. The results suggest that a classifier is less successful in distinguishing images with and without artefacts after exchanging only a small fraction at the fringe of *k*-space. The bigger the *k*-space fraction to be exchanged becomes, the more accurate the results of the classification are. The closer the portion to be exchanged comes to the center of *k*-space, where the low frequencies and the major part of the energy are located, the better detectable the effects of the inconsistencies become. The results show that even if only a single *k*-space row is inconsistent with the rest of *k*-space and it is located in the 80% of lines that are closest to the center of *k*-space, a classification accuracy of around 96.9% or more can be achieved. On the contrary, if a large fraction of *k*-space acquired at the beginning of the scan causes inconsistencies, a classification accuracy of 95.3% can be achieved.

**Figure 4:**
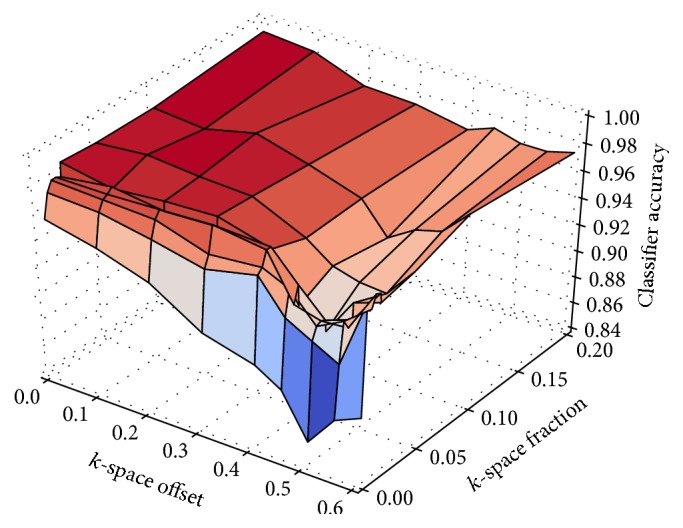
Classification accuracy compared to the position in the acquisition when movement began (offset) and the number of radial spokes (fraction) that were exchanged in *k*-space.

Figure 4 shows the classification accuracy of motion artefacts with a radial sampling trajectory. The classification score usually achieves more than 95% if more than a single radial spoke is causing inconsistencies. In contrast to the results from a Cartesian acquisition, exchanging the first two or more radial spokes of the acquisition can be detected reliably—probably due to the radial sampling pattern where each spoke has an impact on the low frequency content.

However, there is an unexpected valley when inconsistencies occur after 50% of the acquisition has been completed. One explanation for these surprising measurements might be that inconsistent spokes at this particular time lead to smearing in the horizontal direction, whereas inconsistencies at other times tend to smear into a more diagonal or even vertical direction. It seems that the classifier learns to give higher weight to features that are located in the background region. Most of this background region can be found below the object in the region of interest, while there is hardly any background on the left and on the right of the object where smearing can clearly be seen at this particular time point (see Figure 6).

**Figure 5:**
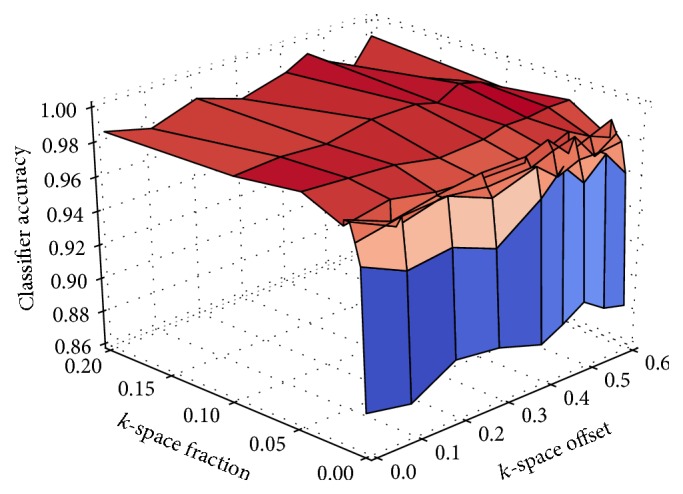
Classification accuracy compared to the position in the acquisition when movement began (offset) and the number of spirals (fraction) that were exchanged in *k*-space.

**Figure 6:**
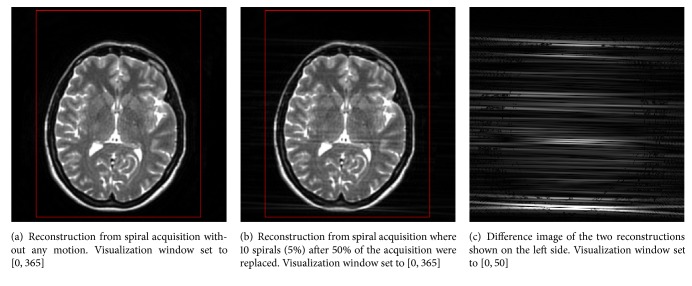
If bulk patient motion occurred after 50% of a radial acquisition had completed, the image quality is degraded by smearing in the horizontal direction. The red frame indicates the region of interest in which features were extracted.

In Figure 5, the classification accuracy on images from a spiral acquisition is compared to the number of spirals that were exchanged in *k*-space and their position in the acquisition. It is evident that having only a single inconsistent spiral cannot be detected as reliably as having more than one inconsistent spiral. This might be due to the spiral acquisition strategy that is more robust to subject movement and can hence suppress slight inconsistencies by design. Note that the classification accuracy does not depend on the position of the spirals to be replaced during the acquisition since each spiral covers approximately the same amount of energy in *k*-space.

Unfortunately, it is hard to compare the results to the study by Mortamet et al. [9]. Since the scans for their study were acquired at various sites with varying hardware, these scans might show apparent differences, for example, different intensity ranges, and unapparent differences, for example, different noise distributions, due to the scanner hardware. A machine learning algorithm would need to be trained very carefully on this dataset in order not to unintentionally learn the characteristics of different scanner systems.

### 3.3. Examples of Respiratory Motion Artefacts

In addition to bulk motion, the effects of periodic motion were investigated on cardiac data. Motion artefacts were introduced as described in Section 2.3.

**Figure 7:**
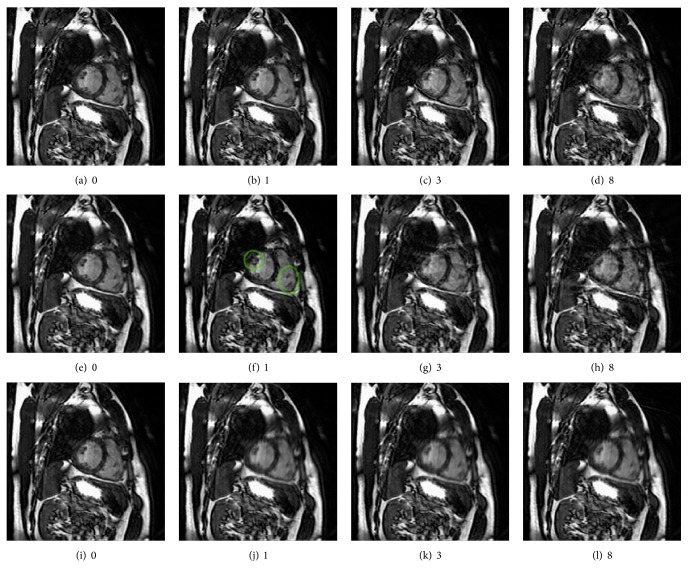
Effects of respiratory motion on the heart. (a), (b), (c), and (d) show scans from a Cartesian acquisition, (e), (f), (g), and (h) from a radial acquisition, and (i), (j), (k), and (l) from a spiral acquisition. The captions indicate the number of breathing cycles that the subjects completed during the acquisition, exposing different levels of artefacts.

In the scans displayed in Figure 7, the synthetic subjects completed different numbers of breathing cycles in a single acquisition as indicated by the captions.  Figure 7(a) was acquired without any motion and shows the left ventricle and the right ventricle. When the subject completed one breathing cycle during the acquisition as seen in Figure 7(b), small structures already started to deform. With more motion, the boundary of the moving region starts to show up as waves of lines around the heart. These streaks also start to decrease the visibility of both ventricles, eventually looking like a layer of noise. Small structures become hard to recognize with more movement.

The scans displayed in Figures 7(e), 7(f), 7(g), and 7(h) simulate the same movement but were acquired with a radial sampling pattern. Compared to the scan without motion, in Figure 7(f), the small dark structures on the left of the right ventricle and on the right of the left ventricle have changed in shape (green ellipse). Then streaking artefacts started to form, showing up as tangential streaks on the edges of both ventricles. These streaks seem to be prominent in regions of high intensities.

In contrast to the two other sampling patterns, movement in a spiral acquisition results in heavy blurring of the moving region. Nevertheless, the amount of blurring seems to remain similar despite of the increasing amount of movement.

### 3.4. Classification Results of Respiratory Motion Artefacts

The evaluation comprised all four types of features, including box-, line-, histogram-, and texture-based features. A classifier was trained for all types of features individually and on feature spaces concatenating histogram and texture features only as well as all four feature types.

**Figure 8:**
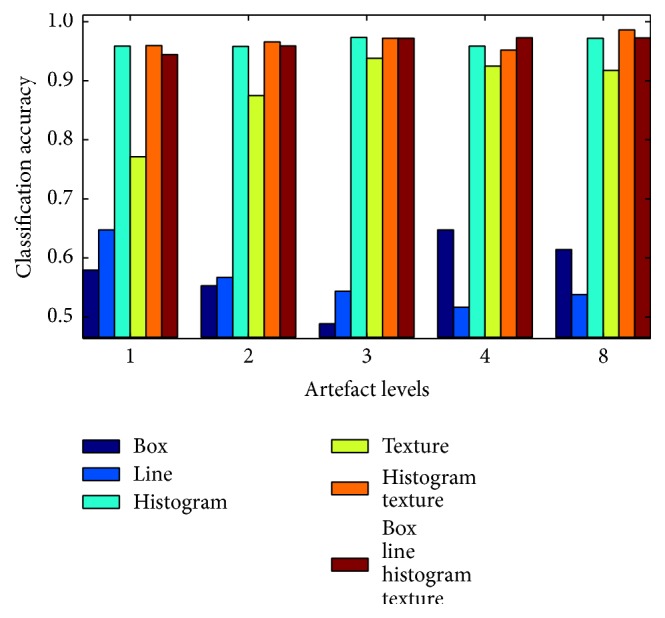
Classification accuracy for different levels of artefacts with a Cartesian acquisition strategy. The artefact levels refer to the number of breathing cycles that a subject completed during the acquisition.

Figure 8 shows the classification accuracy for images from a Cartesian acquisition with different levels of artefacts, which refer to the number of breathing cycles that were squeezed into one acquisition.

The classification accuracy of box features ranges between 48.9% for artefact level 3 and 64.8% for artefact level 4. In general, they do not seem to produce reliable results, firstly because of the low classification accuracy and secondly since there is no obvious relation to the level of artefacts, as one would expect the classification accuracy to rise with higher strength of artefacts. One reason for the modest success rate with box features might be that they are not invariant to rotation, while the subjects were scanned from different angles and therefore their heart appears from different orientations. Similar considerations apply to the line features.

In contrast to box and line features, histogram features achieved a classification accuracy between 95.9% and 97.3%. A part of these histogram-based features was computed on the whole region of interest and is therefore invariant to rotation. The classification accuracy for texture features increased from 77.1% for the lowest level of artefacts to 91.8% for the highest level. A combination of histogram and texture feature spaces as well as a combination of all feature spaces achieved similar results to the histogram features.

**Figure 9:**
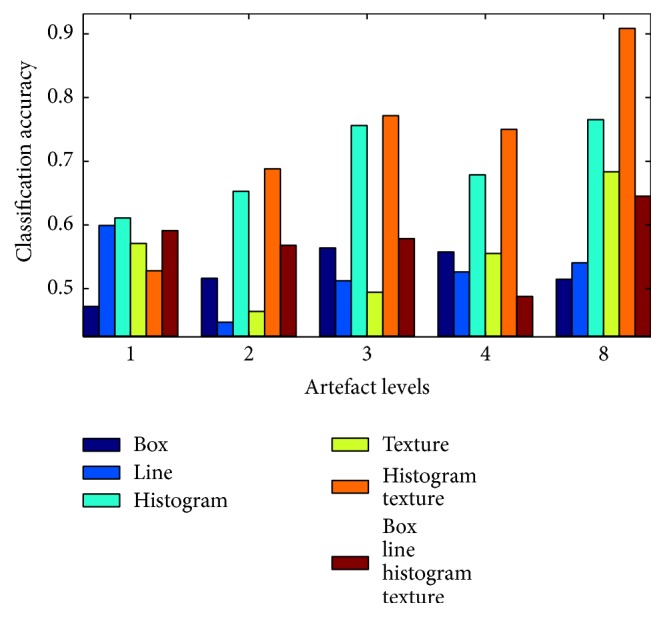
Classification accuracy for different levels of artefacts with a radial acquisition strategy.

The detection rate drops for images acquired with a radial sampling pattern (see Figure 9). The fact that no feature is capable of achieving more than 61.1% (histogram features) for the first level of artefacts is probably the fault of the sampling pattern that is suppressing artefacts by design. As soon as the motion becomes more dominant, particularly histogram-based features seem to detect the increasing level of streaking artefacts that the radial sampling pattern exposes. Combining histogram and texture features outperforms the classification accuracy of a single feature type, achieving an accuracy of 68.8% of correctly classified images at artefact level 2 up to 90.9% at artefact level 8.

**Figure 10:**
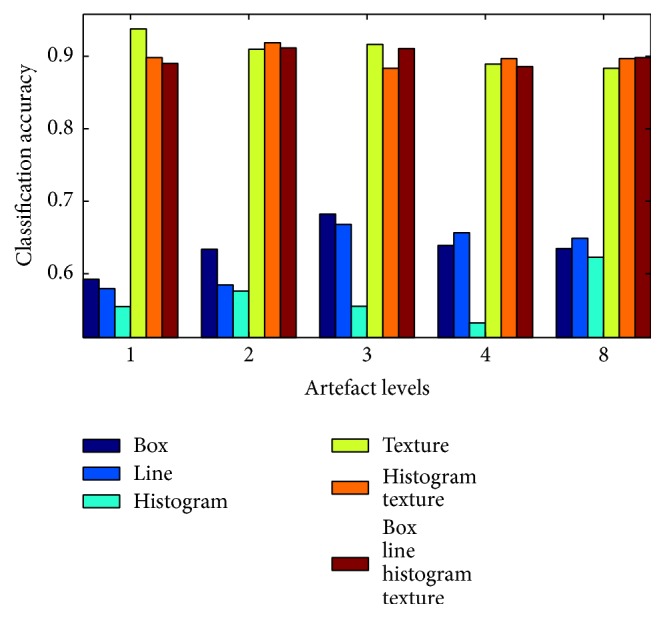
Classification accuracy for different levels of artefacts with a spiral acquisition strategy.

As it can be seen in Figure 10, the histogram features, which worked as the most reliable type of features, fall behind on classifying artefacts from images acquired with a spiral sampling pattern. The classification accuracy of histogram features ranges between 53.2% and 62.2% close to a success rate of pure guessing and they are surpassed by box and line features. However, the texture features succeeded by far, achieving between 88.3% on images of artefact level 8 and 93.8% on images of artefact level 1. Combining texture features with histogram features and the other feature types yields similar detection rates. There is no obvious reason why the performance on fewer artefacts is slightly better than that on more obvious artefacts.

Although the spiral sampling pattern has been developed to be more robust to movement similar to the radial sampling pattern, it is not surprising that already the lowest level of motion can be detected with a success rate of more than 90% after looking at the examples in Figures 7(i), 7(j), 7(k), and 7(l). The spiral sampling pattern starts blurring the region of interest already after little motion occurred. From a visual impression, the amount of blurring does not change much if more than one breathing cycle occurs during one acquisition, supporting the mostly constant detection rate over the different levels of artefacts.

## 4. Conclusion

This work evaluated the automatic detection of motion artefacts using decision forests based on features that have been employed in several MRI studies before. Being able to detect motion from the acquired data would allow a clinician to have real-time feedback on whether the scan should be repeated due to insufficient image quality while the patient is still in the scanner. With the presented approach to simulate realistic motion in *k*-space, we showed how the effects of motion manifest themselves in ghosting and blurring artefacts. Both effects could be found in images from a Cartesian acquisition. With a radial acquisition, the image structures were found to be much sharper, though streaking artefacts became apparent. The radial sampling pattern also showed its robustness to little motion inconsistencies. The effects of motion on images from a spiral acquisition turned up as blurring in the moving regions.

In case of Cartesian sampling, the detectability of inconsistencies was clearly found to be related to the number of lines affected and the time during the acquisition when motion occurred. Artefacts become better detectable when more lines are affected by motion and the closer these lines are to the center of *k*-space, where the low frequencies and most of the energy are located. On scans acquired with a radial and spiral acquisition bulk motion artefacts were found to be detectable with high confidence unless only a very small number of radial spokes or interleaved spirals were affected. It is worth noting that, in case of a radial or spiral sampling pattern, the time point at which the motion occurs in the acquisition does not have an impact on the classification accuracy, since each radial spoke or spiral covers approximately the same amount of energy in *k*-space.

The effects of respiratory motion were evaluated on cardiac scans. In particular histogram-based features led to decent results on images acquired with a radial sampling geometry, and texture features performed well on images acquired with a spiral sampling geometry. Combining the feature spaces of histogram and texture features yielded encouraging results as well.

For future work, to overcome problems with recordings from different orientations, one could use SIFT to produce features that are invariant to image scaling, translation, and rotation and robust to illumination changes [22]. While large key points were shown to perform well on images that had been degraded by blurring and noise, it is not clear how ghosting might affect the matching.

Of course it would be beneficial to evaluate the performance of the machine learning approach developed in this work on real, annotated data to support the conclusions of this paper.

## References

[1] Forman C., Grimm R., Hutter J. M., Maier A., Hornegger J., Zenge M. O. Free-breathing whole-heart coronary MRA: motion compensation integrated into 3D cartesian compressed sensing reconstruction.

[2] Wetzl J., Forman C., Wintersperger B. J. (2016). High-resolution dynamic CE-MRA of the thorax enabled by iterative TWIST reconstruction. *Magnetic Resonance in Medicine*.

[3] Zaitsev M., Maclaren J., Herbst M. (2015). Motion artifacts in MRI: a complex problem with many partial solutions. *Journal of Magnetic Resonance Imaging*.

[4] Hutter J., Schmitt P., Saake M. (2015). Multi-dimensional flow-preserving compressed sensing (MuFloCoS) for time-resolved velocity-encoded phase contrast MRI. *IEEE Transactions on Medical Imaging*.

[5] Paudyal S. Artefacts in MRI. http://www.slideshare.net/SudilPaudyal/mri-artifacts.

[6] Wetzl J., Forman C., Maier A., Hornegger J., Zenge M. O. Prediction of the benefit of motion-compensated reconstruction for whole-heart coronary MRI.

[7] Welch E. B., Felmlee J. P., Ehman R. L., Manduca A. (2002). Motion correction using the k-space phase difference of orthogonal acquisitions. *Magnetic Resonance in Medicine*.

[8] Drobnjak I., Gavaghan D., Süli E., Pitt-Francis J., Jenkinson M. (2006). Development of a functional magnetic resonance imaging simulator for modeling realistic rigid-body motion artifacts. *Magnetic Resonance in Medicine*.

[9] Mortamet B., Bernstein M., Jack C., Thiran J.-P., Krueger G. (2011). Multi-slice 2D MR imaging: automatic assessment of image quality. *Journal of Magnetic Resonance Imaging*.

[10] Elster A. D. Motion artifacts direction - questions and answers in MRI. http://mri-q.com/motion-artifact-direction.html.

[11] Forman C., Aksoy M., Hornegger J., Bammer R. (2011). Self-encoded marker for optical prospective head motion correction in MRI. *Medical Image Analysis*.

[12] Biomedical Image Analysis Group: Imperial College London Ixi - information extraction from images. http://brain-development.org/ixi-dataset/.

[13] Wetzl J., Stalder A. F., Schmidt M., Ourselin S., Joskowicz L., Sabuncu M. R., Unal G., Wells W. (2016). Joint estimation of cardiac motion and T_1_^*^ maps for magnetic resonance late gadolinium enhancement imaging. *Lecture Notes in Computer Science*.

[14] Mendis S., Puska P., Norrving B. (2011). Global atlas on cardiovascular disease prevention and control. *World Health Organization in collaboration with the World Heart Federation and the World Stroke Organization*.

[15] Suinesiaputra A., Cowan B. R., Al-Agamy A. O. (2014). A collaborative resource to build consensus for automated left ventricular segmentation of cardiac MR images. *Medical Image Analysis*.

[16] McLeish K., Hill D. L. G., Atkinson D., Blackall J. M., Razavi R. (2002). A study of the motion and deformation of the heart due to respiration. *IEEE Transactions on Medical Imaging*.

[17] Bai W., Shi W., de Marvao A. (2015). A bi-ventricular cardiac atlas built from 1000+ high resolution MR images of healthy subjects and an analysis of shape and motion. *Medical Image Analysis*.

[18] Atkinson D., Hill D. L., Stoyle P. N., Summers P. E., Keevil S. F. (1997). An autofocus algorithm for the automatic correction of motion artifacts in MR images. *Lecture Notes in Computer Science*.

[19] Boland M. V. Quantitative description and automated classification of cellular protein localization patterns in fluorescence microscope images of mammalian cells. http://murphylab.web.cmu.edu/publications/boland/boland_node26.html.

[20] Haralick R. M., Shanmugam K., Dinstein I. (1973). Textural features for image classification. *IEEE Transactions on Systems, Man and Cybernetics*.

[21] Müller K., Maier A. K., Schwemmer C. (2014). Image artefact propagation in motion estimation and reconstruction in interventional cardiac C-arm CT. *Physics in Medicine and Biology*.

[22] Lowe D. G. (2004). Distinctive image features from scale-invariant keypoints. *International Journal of Computer Vision*.

